# Complete genome sequences of *Thermus thermophilus* strains isolated from Shirahama Hot Spring in Japan

**DOI:** 10.1128/mra.00316-24

**Published:** 2024-07-11

**Authors:** Yuki Takafuji, Tim Fischer, Kentaro Miyazaki, Kohsuke Honda

**Affiliations:** 1 International Center for Biotechnology, Osaka University, Suita, Osaka, Japan; 2 Industrial Biotechnology Initiative Division, Institute for Open and Transdisciplinary Research Initiatives (OTRI), Suita, Osaka, Japan; SUNY College of Environmental Science and Forestry, Syracuse, New York, USA

**Keywords:** thermophiles, *Thermus thermophilus*, hot springs, genome analysis, megaplasmids

## Abstract

We isolated six *Thermus thermophilus* strains from Shirahama Hot Spring in Japan. Complete genome sequences, determined by combining Oxford Nanopore long-read and Illumina short-read sequence data, revealed that they showed >99.9% average nucleotide identities with each other and approximately 97% to the genome of the type strain HB8^T^.

## ANNOUNCEMENT

An extremely thermophilic bacterium *Thermus thermophilus* was first isolated by Tairo Oshima from Mine Hot Spring in Japan in 1968 (1, 2). Since then, many *T. thermophilus* strains have been isolated from hot springs in Japan, Mine (3 – 5), Arima (6, 7), and Senami (8), as well as from thermal areas worldwide (9) (10). As a model thermophilic organism (11), researchers have intensively studied the bacterium biochemically (12 – 15), structurally (16 – 18), and genetically (15, 19 – 21). Most of these studies have been conducted using the representative strains such as HB8 and HB27, but recent trends in this organism are shifting to genomic studies of novel isolates (3 – 8, 22, 23). To date, 25 complete genome sequences have been reported for *T. thermophilus* (https://www.ncbi.nlm.nih.gov/genome/browse/#!/prokaryotes/461/).

We collected hot water samples at Shirahama Hot Spring (33.6772 N 135.3383 E) in Japan. Samples were spread over Lennox LB [1% (wt/vol), 0.5% (wt/vol) yeast extract, and 0.5% (wt/vol) NaCl] agar plates [1.6% (wt/vol)]. After cultivation at 70°C overnight, dozens of well-separated single colonies were isolated. The 16S rRNA genes were analyzed by the colony PCR method (21), and all showed 99.5% identity to *T. thermophilus* HB8 (NR_037066.1). We randomly selected six strains and subjected to whole-genome analysis.

To prepare genomic DNA, cells were grown in 2 mL of LB at 70°C for 16 h with vigorous shaking (200 rpm). Genomic DNA was purified using a Wizard Genomic DNA Purification Kit (Promega). For long-read sequencing, unsheared genomic DNA (1 µg) was treated with a Short-Read Eliminator Kit (Circulomics) to remove fragments <10 Kbp, and a library was constructed using a Ligation Sequencing Kit [Oxford Nanopore Technologies (ONT)]. Sequencing was performed with a MinION system on a FLO-MIN106 flow cell (ONT). Base calling was conducted using Guppy v.6.5.7. For short-read sequencing, the DNA PCR-Free Prep Kit (Illumina) was used to generate paired-end libraries with approximately 450 bp inserts. Sequencing was performed using a MiSeq Reagent Kit v.2 (300 cycles) with 251 bp reads. These raw sequencing data were adapter trimmed using cutadapt v.1.9.2 (24). Trimmed long- and short-read data were assembled using flye v.2.9.1-b1780 (25), and the assembly was polished using Pilon v.1.23 (26). Default parameters were used except where otherwise noted.

All strains contained a single chromosome and one (TF19, TF24, TF25, TF27, and TF28) or two (TF21) plasmids. Automatic annotation was conducted using DFAST v.1.6.0 (27); genomic features are summarized in Table 1. A JSpecies analysis (28) revealed that the genome sequences of these strains had average nucleotide identities (ANIs) of ≥99.9% to each other with ≥95% aligned sequence. MiGA TypeMat search (29) for the closest genome sequence revealed that all the strains showed the highest identities to strain HB8 (NZ_AP024984), the type strain of this species, with 97% ANIs with the fraction of genome shared of 83%.

**TABLE 1:** Sequencing metrics for the six *T. thermophilus* strains in this study

Strain	MinION (long read)					MiSeq (short read)				Complete genome				
	No. of reads	N_50_ (b)	Total length (Mb)	SRA accession no.	Average read depth (×)	No. of paired-end reads	Total length (Mb)	SRA accession no.	Average read depth (×)	Length (bp)	GC content (%)	Average read depth (×)	No. of coding sequences	GenBank accession no.
TF19	108,907	47,741	699.5	DRR536140	309.8	377,226	189.4	DRR536141	83.9					
Chromosome										1,921,656	69.1	77.8	2,069	AP031323
pTF19b										336,643	68.0	91.7	370	AP031324
														
TF21	97,921	56,584	655.5	DRR536142	291.2	425,645	213.7	DRR536143	94.9					
Chromosome										1,920,958	69.1	86.9	2,062	AP031325
pTF21b										303,403	68.0	92.8	332	AP031326
pTF21c										26,522	67.2	65.1	29	AP031327
														
TF24	75,613	60,118	565.6	DRR536144	256.9	351,834	176.6	DRR536145	80.2					
Chromosome										1,922,100	69.1	75.4	2,064	AP031328
pTF24b										279,478	67.9	76.8	307	AP031329
														
TF25	124,457	53,857	757.6	DRR536146	335.7	376,849	189.2	DRR536147	83.8					
Chromosome										1,919,884	69.1	77.3	2,059	AP031330
pTF25b										336,644	68.0	90.8	371	AP031331
														
TF27	128,536	51,747	807.9	DRR536148	365.1	419,473	210.6	DRR536149	95.2					
Chromosome										1,922,104	69.1	88.6	2,067	AP031332
pTF27b										290,779	68.0	96.9	326	AP031333
														
TF28	91,554	55,231	591.75	DRR536150	262.2	347,569	174.5	DRR536151	77.3					
Chromosome										1,920,956	69.1	72.4	2,058	AP031334
pTF28b										335,479	68.0	78.5	366	AP031335
														

## Data Availability

All the *T. thermophilus* strains reported in this paper are associated with BioProject PRJDB17653. The genome sequences and raw sequencing data are available under the accession numbers listed in Table 1.
